# Knowledge Regarding Hypertension and Amount of Diet Consumption Among Adults From Uttarakhand: A Comparative Survey

**DOI:** 10.7759/cureus.39065

**Published:** 2023-05-15

**Authors:** Neetu Kataria, Vasantha C Kalyani, Sonia Gulia, Kaleeswari G

**Affiliations:** 1 Neurosciences Nursing, All India Institute of Medical Sciences, Rishikesh, IND; 2 Medical Surgical Nursing, All India Institute of Medical Sciences, Deoghar, IND; 3 Medical Surgical Nursing, All India Institute of Medical Sciences, Rishikesh, IND

**Keywords:** hypertension, public health, risk factors, urban and rural community, knowledge assessment, diet consumption

## Abstract

Background: Hypertension is becoming more common worldwide, and by 2025, its incidence is predicted to rise by 32.4%. The present study aims to assess the knowledge related to hypertension and the amount of diet consumption among adults at risk of developing hypertension in both rural and urban areas of Uttarakhand.

Methods: A cross-sectional survey was conducted on 667 adults at risk of hypertension. The study sample included adults selected from the rural and urban communities of Uttarakhand. A semi-structured questionnaire on knowledge regarding hypertension and self-reported amount of diet consumption was used as a tool for data collection.

Results: The mean age of participants in this study was 51.46 ± 1.44 years, and the majority of the participants had poor knowledge regarding hypertension as a disease and its consequences as well as preventive measures. The mean days for consumption of fruits were three days, four days for green vegetables, two days for eggs, and two days for a well-balanced diet, and the mean ± SD of a non-vegetarian diet consumption was 128 ± 182 gm. Another highlighted significant mean difference was found between knowledge of raised blood pressure with the amount of consumption of fruits, green leafy vegetables, a non-vegetarian diet, and a well-balanced diet.

Conclusion: In the present study, knowledge of blood pressure and raised blood pressure with its related factors was poor among all participants. The average consumption of all types of diet was two to three days a week, which was borderline, based on recommended dietary allowances. The knowledge related to raised blood pressure and its associated factors had significant mean differences for the mean consumption of fruits, non-vegetarian diet, and well-balanced diet.

## Introduction

The global impact of hypertension on people's quality of life and the resources of the healthcare system is enormous [1]. Hypertension is becoming more common worldwide, and by 2025, its incidence is predicted to rise by 32.4% [2]. Increased mortality and risk of cardiovascular disorders, such as myocardial infarction, angina pectoris, heart failure, and stroke, are both a result of hypertension. It is one of the major factors contributing to the global burden of disease and early mortality [1-3]. The World Health Organization estimates that there are 600 million people with high blood pressure globally, and 7.14 million fatalities are thought to be directly related to hypertension each year [4]. Since the 20th century, blood pressure has increased in Sub-Saharan Africa, Oceania, and East, South, and Southeast Asia as compared to high-income countries. Systolic blood pressure (SBP) of more than 115 mmHg was projected to be the cause of 8.5 million fatalities in 2015, 88% of which occurred in low- and middle-income nations [5]. Only 21% of hypertension individuals worldwide in 2021 had their blood pressure under control [6].

India, a lower middle-income nation with a population of more than one billion (rural population: 68.84%), is experiencing a rapid epidemiologic transition characterized by an increased prevalence of non-communicable diseases. Hypertension is a significant health issue in both urban and rural areas of India [7,8]. Urban environments are more common than rural ones because of factors such as a lack of physical activity, unhealthy food consumption, crowding, extensive use of motor vehicles, and poor air quality [2,9].

Modifiable risk factors include smoking, eating poorly (inadequate diet taken as per recommended dietary allowance (RDA)), being inactive (sedentary lifestyle), and drinking excessively (intake of alcohol). In addition, there is a strong link between general and central obesity and hypertension [10]. According to Indian hypertension 2013 guidelines, SBP at or above 130-139 mmHg and diastolic blood pressure (DBP) at or above 90 mmHg are considered to be signs of hypertension [11].

The relationship between food and health is complex. More than 2,500 years ago, Hippocrates said: “Let food be thy medicine and medicine be thy food.” A healthy diet can optimize both short- and long-term health and can help reduce the risk for many health conditions [12]. Dietary sodium and fats, including trans fats and saturated fats, are associated with the elevation of blood pressure, while dietary potassium and poly-unsaturated fats lower the risk of hypertension and stroke. Regular and frequent consumption of fruits and vegetables can help prevent high blood pressure, congenital heart disease, and stroke [3]. The present study's objective was to assess the knowledge related to hypertension among adults from both rural and urban areas of Uttarakhand. Another objective was to assess the pattern of diet consumption and its association with knowledge related to hypertension among adults at risk of hypertension in Uttarakhand.

## Materials and methods

Study design and setting

A cross-sectional comparative survey was conducted on adults residing in rural and urban communities of Uttarakhand, India.

Study participants

The study sample included human adults aged 18-40 years. The inclusion criteria were adults residing in rural and urban areas of Uttarakhand, with normotensive (120-129/80 mmHg) as well as hypertensive status with SBP/DBP > 130-139/80-89 mmHg (as per Indian hypertension guidelines 2013), and adults taking antihypertensive treatment.

Sample size

The sample size was calculated to be 667 by using the WinPepi® program to estimate a mean confidence level of 95%, with an acceptable difference of 40 per 1000, an assumed rate of 500 per 1000 (taken from rural and urban areas of Uttarakhand, India by using this WinPepi® model), and expected loss of subjects of 10%.

Sampling technique

The study sample was selected by using a purposive sampling technique.

Quantitative variables

Outcomes were knowledge regarding hypertension (frequency and percentage), diet consumption (mean ± SD), and mean difference ± SD between knowledge questionnaire.

Operational definition

Amount of Diet Consumption

It refers to the specific combination and quantity of food that is regularly consumed by an individual or population.

Well-Balanced Diet

A well-balanced diet is defined according to RDA guidelines, as the amount of diet, energy, and nutrition required to maintain a healthy body.

Data sources/measurement

A semi-structured questionnaire regarding knowledge about hypertension and self-reported amount of diet consumption was used as a tool for data collection. The data collection tool consisted of three parts: part 1 for sociodemographic data, part 2 for assessing the knowledge regarding hypertension, and part 3 for assessing the pattern of diet consumption. The tool was validated by five experts from nursing and community & family medicine. The reliability of the tool was measured as 0.78 by Cronbach’s alpha.

Data collection procedure

Patients were selected by using the purposive sampling technique. Informed consent was taken from each subject to enroll in the present study. A semi-structured questionnaire having two sections was used for data collection for one year (September 2018-2019) after getting approval from the Institutional Ethical Committee (IEC), All India Institute of Medical Sciences, Rishikesh (IEC/IM/RC69/2016/07; letter provided in August 2016). The first section consists of the socio-demographic variables of the participants whereas the outcomes reported in the second section consist of questions related to knowledge regarding hypertension and pattern of diet consumption.

Statistical analysis

The data were entered into Microsoft Excel version 13.0 (Microsoft Corporation, Redmond, WA) and analyzed using SPSS version 23.0 software (IBM Corp., Armonk, NY). The study participant’s age and consumption of diet were recorded in the form of mean ± SD while categorical variables were denoted by frequency distribution; the differences between knowledge regarding hypertension and pattern of diet consumption were analyzed by the Mann-Whitney U test. For all variables, p-values ≤ 0.05 were considered significant.

## Results

Study description

Participants

The total sample size was 667 taken with a 100% response rate. The mean age of the whole group of participants was 51.46 ± 1.44 years, which was found as normally distributed. The results were divided into three sections, including baseline distribution of socio-demographic characteristics, knowledge regarding hypertension, and the pattern of diet consumption. The mean difference between the tools was analyzed by using the Mann-Whitney U test.

Specification of the data

Table 1 shows the baseline distribution of sociodemographic variables among 667 adults with the mean age of participants being 51.46 ± 1.44 years. The majority of the participants were females, belonged to nuclear families, were educated up to graduation, and had jobs in their hands.

**Table 1:** Baseline distribution of the socio-demographic characteristics among adults (N = 667)

S. No.	Variable	Category	Frequency (%)
1.	Age (in years)	Mean ± SD	51.46 ± 1.44
2.	Gender	Male	313 (46.9)
		Female	354 (53.1)
3.	Type of family	Nuclear	418 (62.7)
		Joint	214 (32.1)
		Extend	35 (5.2)
4.	Education	Postgraduate	121 (18.1)
		Graduate	165 (24.7)
		Intermediate	127 (19)
		High school	81 (12.1)
		Junior high school	71 (1.6)
		Primary school	32 (4.8)
		Illiterate	70 (10.5)
5.	Employment	Job	263 (39.4)
		Unemployed	38 (5.7)
		Student	61 (9.1)
		Retired	1 (1)
		Housewife	201 (30.1)
		Self-employed	62 (9.3)
		Labor	41 (6.1)

Outcome data and main results

Table 2 shows the frequency distribution of knowledge related to hypertension among adults. The majority of the participants did not consider hypertension as a disease and they did not know about the consequences of the uncontrollable rise in blood pressure. The majority of the participants did not know about the total duration for taking antihypertensives and which habits can prevent hypertension. The majority of the participants knew that salt in food is related to blood pressure. It can be interpreted that the majority of the participants had poor knowledge related to blood pressure except for salt consumption in the diet.

**Table 2:** Frequency distribution of knowledge related to blood pressure (N = 667)

S. No.	Variable	Category	f	%
1.	Do you know why high blood pressure is considered a disease?	Yes	279	41.8
		No	388	58.2
2.	Do you know what can happen if blood pressure remains uncontrolled?	Yes	248	37.2
		No	419	62.8
3.	Do you know how long blood pressure medication needs to be taken?	Yes	173	25.9
		No	494	74.1
4.	What habits can help prevent high blood pressure?	Yes	240	36
		No	427	64
5.	Is the amount of salt in food related to high blood pressure?	Yes	372	55.8
		No	295	44.2

Table 3 shows the mean consumption of diet patterns in days in a week. The total days for food consumption by all participants were as follows: fruits for three days, green vegetables for four days, eggs for two days, and a well-balanced diet for two days, with a mean of 129.6 gm of non-vegetarian diet in a week. It can be interpreted that the average days of consumption of diet were two to three days in a week, which is quite low in terms of RDA for the respective food items listed below.

**Table 3:** Mean distribution of the pattern of diet consumption (N = 667)

S. No.	Variable (in days)	Mean ± SD
1.	How many pieces of fruit do you eat in a week?	2.79 ± 2.04
2.	How many servings of green vegetables do you eat in a week?	3.74 ± 1.98
3.	How many eggs do you eat in a week?	2.04 ± 2.49
4.	How much non-vegetarian food do you eat in a week (in grams)?	128.65 ± 182.6
5.	How many days a week do you eat a well-balanced diet for weight loss?	2.19 ± 0.97

Table 4 shows that the knowledge of raised blood pressure can significantly differ in the mean consumption of diet for fruits, non-vegetarian diet, and well-balanced diet in a week (p < 0.01). Consecutively, knowledge related to the consequences of uncontrolled blood pressure had a significant difference in the mean consumption of fruits and a well-balanced diet in a week (p < 0.01). In continuation, the knowledge of the duration for which antihypertensive treatment should be taken was significantly different in the mean of non-vegetarian diet and well-balanced diet consumption in a week (p < 0.01). Another question about the knowledge related to habits that can prevent the rise in blood pressure had a significant difference in the mean consumption of fruits and a well-balanced diet in a week (p < 0.01). Similarly, the question of knowledge of the relation of salt consumption with blood pressure was significantly different in the mean consumption of fruits and green leafy vegetables in a week (p < 0.01).

**Table 4:** Mean difference between having knowledge of hypertension and pattern of diet consumption (N = 667) Note: Mann-Whitney U test; * p-value < 0.05 considered as significant.

S. No.	Knowledge of hypertension and pattern of diet questions	Z value (p-value)	95% CI (lower; upper)
Do you know why high blood pressure is considered a disease?			
1.	How many pieces of fruit do you eat in a week?	2.65 (0.008*)	(0.10; 0.71)
2.	How much non-vegetarian food do you eat in a week (in grams)?	-3.388 (0.001*)	(-78.18; -19.49)
3.	How many days a week do you eat a well-balanced diet for weight loss?	-4.899 (0.001*)	(-0.51; -0.220)
Do you know what can happen if blood pressure remains uncontrolled?			
1.	How many pieces of fruit do you eat in a week?	2.932 (0.003*)	(0.15; 0.77)
2.	How many days a week do you eat a well-balanced diet for weight loss?	-5.419 (0.001*)	(-0.56; -0.26)
Do you know how long blood pressure medication needs to be taken?			
1.	How much non-vegetarian food do you eat in a week (in grams)?	-2.360 (0.019*)	(-69.29; -6.35)
2.	How many days a week do you eat a well-balanced diet for weight loss?	-5.024 (0.0010*)	(-0.59; -0.26)
What habits can help prevent high blood pressure?			
1.	How many pieces of fruit do you eat in a week?	2.522 (0.012*)	(0.08; 0.71)
2.	How many days a week do you eat a well-balanced diet for weight loss?	-4.716 (0.001*)	(-0.51; -0.21)
Is the amount of salt in food related to high blood pressure?			
1.	How many pieces of fruit do you eat in a week?	3.177 (0.002*)	(0.18; 0.79)
2.	How many servings of green vegetables do you eat in a week?	2.452 (0.01*)	(0.07; 0.67)

## Discussion

Cardiovascular diseases (CVDs) are a growing contributor to the burden of disease worldwide, with epidemics of CVD spreading to many areas of the world that are going through a rapid change in their health [3]. According to Kumar et al., the number of chronic disease-related deaths in India increased from 3.78 million (40.4%) in 1990 to 7.63 million (66.7%) in 2020 [8].

Studies conducted on high-altitude natives of the Andes, Tibetans of India, and rural high-altitude natives of the Greater Himalayas have revealed that the blood pressure values of the natives are lower than those who live in plains and at low altitudes [13].

Diet and nutrition have been extensively investigated as risk factors for major CVDs like coronary artery disease and stroke and are also linked to other cardiovascular risk factors like diabetes, high blood pressure, and obesity [3].

The major study finding was that knowledge related to blood pressure was average to poor, which was supported by previous study findings that knowledge of reasons, consequences, and preventive measures was poor [14]. Another highlighted finding from our study was a significant mean difference found between knowledge of raised blood pressure with the amount of consumption of fruits, green leafy vegetables, a non-vegetarian diet, and a well-balanced diet, which was supported by a previous study that found that poor lifestyle pattern of imbalanced intake of fruits and vegetables was an independent predictor for causing hypertension [14]. Another previous study reported that some cultural and emotional factors also affect knowledge of hypertension and hence behavioral patterns for dietary consumption, which can affect the blood pressure of the participants, which was also found in our study findings, as a correlation was found between knowledge and dietary consumption [15].

Another finding was the total proportion of proper diet consumption in a week was two to three days on average, which was similar to a previous study that suggested that age, sedentary lifestyle, and unhealthful eating habits are only a few of the risk factors that have a high correlation with the development of hypertension and other cardiovascular illnesses [7,8].

One of the best-known dietary strategies for lowering blood pressure is the Dietary Approaches to Stop Hypertension (DASH) pattern, which significantly lowered blood pressure in both normotensive and hypertensive subjects. The DASH diet, which is high in fruits, vegetables, and low-fat dairy products, and low in saturated and total fat, has been shown to be effective in randomized controlled trials in specific populations, including obese people with hypertension and type 2 diabetes and these findings matched the consumption of the DASH diet with the present study findings.

Lifestyle modification strategies alone and in combination with others have great potential to prevent hypertension, often at less cost than current pharmacological interventions [16]. The following non-pharmacological measures are commonly recommended in global guidelines to prevent and reduce the risk of complications associated with hypertension: BMI less than 25 kg/m^2^; increased physical activity; limiting alcohol intake to two drinks per day for men and one drink per day for women; and controlling dietary salt intake to 6 gm/day [17]. These results are also in line with this study's conclusion of restricting salt intake and having a well-balanced diet by excluding excess alcohol intake.

Awareness and knowledge regarding raised blood pressure were important aspects that were studied in our study and were found to be average to poor. Every common man knows excess intake of salt leads to diseases like obesity, heart ailments, and blood pressure. Minimal intake of salt or any nutrient helps us attain good health and prevents the body from contracting any disease [18]. The overall imbalance between unhealthy diet intake and physical activity leads to obesity, which contributes to high blood pressure and high cholesterol. It is a multifactorial disease and changes in the levels of blood pressure and blood lipids differ by age, sex, and race and are influenced by body fat and dietary patterns [19]. The emergence of hypertension can be attributable to population growth and aging, increasing urbanization, inadequate healthcare systems, and lifestyle changes. Among these risk factors, nutritional deficiencies are the main cause of non-communicable diseases. At last, we need to prevent and get control over hypertension among adults by motivating adults to adopt an active lifestyle, follow low salt and oily diet, increase fiber intake in their diet, remain stress-free, and try to maintain a good sleep-wake cycle by performing daily yoga and meditation throughout the year.

Strengths and limitations of the study

The strength of the study is mainly the considerable sample size calculated as an appropriate size to generalize the findings of the study into the community areas. The study design could have been experimental design, and a control group can be taken as part of the limitations of the study.

## Conclusions

The present study concluded that knowledge of blood pressure and raised blood pressure with its related factors was average to poor among all participants. The average consumption of all types of diet was two to three days a week, which was borderline according to the RDA. The knowledge related to raised blood pressure and its factors had significant mean differences for the main diet consumption of fruits, non-vegetarian diet, and well-balanced diet. The researchers recommended that there is a need to conduct some interventional strategies to improve community participants’ knowledge regarding raised blood pressure, especially those underserved by healthcare services in Sub-Himalayan regions. Few clinical trials can be conducted to assess the effect of audio-visual aids and teaching-learning interventions upon knowledge and practice to prevent raised blood pressure and its lethal complications.
